# Detecting Coevolution in and among Protein Domains

**DOI:** 10.1371/journal.pcbi.0030211

**Published:** 2007-11-02

**Authors:** Chen-Hsiang Yeang, David Haussler

**Affiliations:** 1 Simons Center for Systems Biology, Institute for Advanced Study, Princeton, New Jersey, United States of America; 2 Center for Biomolecular Science and Engineering, University of California Santa Cruz, Santa Cruz, California, United States of America; University of Chicago, United States of America

## Abstract

Correlated changes of nucleic or amino acids have provided strong information about the structures and interactions of molecules. Despite the rich literature in coevolutionary sequence analysis, previous methods often have to trade off between generality, simplicity, phylogenetic information, and specific knowledge about interactions. Furthermore, despite the evidence of coevolution in selected protein families, a comprehensive screening of coevolution among all protein domains is still lacking. We propose an augmented continuous-time Markov process model for sequence coevolution. The model can handle different types of interactions, incorporate phylogenetic information and sequence substitution, has only one extra free parameter, and requires no knowledge about interaction rules. We employ this model to large-scale screenings on the entire protein domain database (Pfam). Strikingly, with 0.1 trillion tests executed, the majority of the inferred coevolving protein domains are functionally related, and the coevolving amino acid residues are spatially coupled. Moreover, many of the coevolving positions are located at functionally important sites of proteins/protein complexes, such as the subunit linkers of superoxide dismutase, the tRNA binding sites of ribosomes, the DNA binding region of RNA polymerase, and the active and ligand binding sites of various enzymes. The results suggest sequence coevolution manifests structural and functional constraints of proteins. The intricate relations between sequence coevolution and various selective constraints are worth pursuing at a deeper level.

## Author Summary

The sequences of different components within and across genes often undergo coordinated changes in order to maintain the structures or functions of the genes. Identifying the coordinated changes—the “coevolution”—of those components in the context of evolution is important in predicting the structures, interactions, and functions of genes. The authors incur a large-scale screening on all the known protein sequences and build a compendium about the coevolving relations of all protein domains—subunits of proteins. The majority of the coevolving protein domains either belongs to the same proteins, appears in the same protein complexes, or shares the same functional annotations. Furthermore, coevolving positions in the same proteins or protein complexes are spatially coupled, as they tend to be closer than random positions in the 3-D structures of the proteins/protein complexes. More strikingly, many coevolving positions are located at functionally important sites of the molecules. The results provide useful insights about the relations between sequence evolution and protein structures and functions.

## Introduction

Coevolution is prevalent at species, organismic, and molecular levels. At the molecular level, selective constraints operate on the entire system, which often require coordinated changes of its components. The most well-known example is the compensatory substitution of nucleic acid pairs in RNA secondary structures [1–6]. Interacting nucleotides vary between AU, CG, and GU pairs in different species in order to maintain the hydrogen bonds.

Coordinated changes of amino acid residues have also been investigated. Typically these studies acquired one (or two) family(ies) of aligned sequences and examined covariation between aligned positions or of the entire sequences. Some of these have applied different covariation metrics including correlation coefficients [7–8], mutual information [9–13], and the deviance between marginal and conditional distributions [14]. These studies demonstrate that sequence covariation is powerful in detecting protein–protein interactions [7,12], ligand-receptor bindings [7,12], and the folding structure of single proteins [10,13]. In addition to direct physical interactions, distant coevolving amino acid residues are reported to be energetically coupled [14] or subject to the functional constraints of the proteins [8].

A major drawback of many covariation metrics is the lack of phylogenetic information. The sequences manifesting the same level of covariation may arise from either a few independent substitutions in early ancestors or correlated changes along multiple lineages [15,16]. In RNA structure prediction, many authors have thereby extended the continuous-time Markov process (CTMP) of sequence substitution [17] to coevolving nucleic acid pairs [3,4,6,18]. However, direct application of these models to protein coevolution is intractable due to the large number of parameters (a 400 × 400 matrix) in the CTMP of amino acid pairs. This problem was addressed by replacing amino acids in a CTMP with simplified, surrogate alphabet sets such as the presence/absence of a protein in each species [16] or the charge and size of amino acid groups [19]. Yet this simplification deviates from the standard CTMP of sequence substitution, in which a rich set of empirical models are available.

All the previous studies of detecting protein coevolution target a few proteins or protein domains, such as myoglobin [19], PGK [7], Ntr family [12], PDZ domain family [14], Gag, Hsp90, and GroEL proteins [8]. The availability of large-scale protein sequences and their phylogenetic information allows us to perform a systematic screening on all the known protein families. Such large-scale screening will give comprehensive information of coevolution among all the protein domains and provide insight about their physical/functional couplings.

We propose a general coevolutionary CTMP model which requires neither simplification of states nor prior knowledge about interactions, and has only one extra free parameter. Sequence substitution of the two sites is modeled by a continuous-time Markov process. The null (independent) model hypothesizes that two sites evolve independently. The alternative (coevolutionary) model is obtained from the null model by reweighting the independent substitution rate matrix to favor double over single changes. We apply this model to all the inter- and intra-domain position pairs in all the known protein domain families in Pfam database [20]. Strikingly, from a large number of pairwise comparisons the coevolving domain pairs are highly enriched with domains in the same proteins, protein complexes, or possessing the same functions. Moreover, the coevolving positions demonstrate a tendency of spatial coupling and are mapped to functionally important sites of their proteins.

## Results

### Overview of the Coevolutionary Model

We extend the CTMP sequence substitution to model coevolution of amino acid position pairs. The state transitions of a CTMP at an infinitesimal time interval follow a matrix differential equation (Equation 1). The instantaneous transition rates are specified by a 20 × 20 substitution rate matrix *Q*. A CTMP of an amino acid pair is obtained by concatenating the sequence states of two amino acid positions. The substitution rate matrix of two independent amino acid positions can be directly derived from the CTMP of single sites. However, the rate matrix of a general two-component CTMP has much fewer constraints and a larger dimension (400 × 400). We simplify the substitution rate matrix by penalizing all the entries of single changes and rewarding all the entries of double changes with the same weight factors. This coevolutionary model introduces very few extra free parameters, thus it is easy to learn and less vulnerable to overfitting. By applying this general coevolutionary model to RNA sequences, we successfully predicted RNA secondary and tertiary interactions [21].

**Figure 1:**
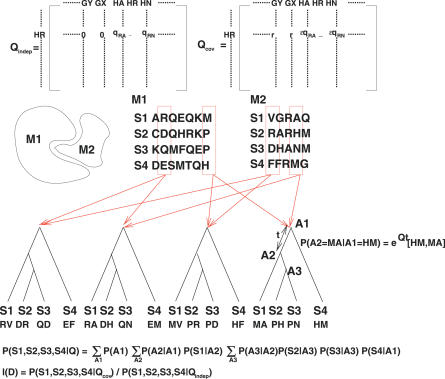
Framework of the Coevolutionary Model (Top row) The independent rate matrix *Q_indep_* is derived from the single amino acid rate matrix. Entries corresponding to both amino acid changes are zeros and entries corresponding to single amino acid changes have the same rates as the single amino acid rate matrix (e.g., *Q_indep_*[*HR*,*HA*] = *Q*[*R*,*A*] ≡ *q_RA_*). The coevolutionary rate matrix *Q_cov_* is obtained by reweighting the independent rate matrix. Entries of single amino acid changes are penalized by multiplying *ɛ* and entries of both amino acid changes are rewarded by replacing zeros with *r*. (Second row) Suppose two protein domains *M*_1_ and *M*_2_ interact at certain positions. We acquire the homologous domains of *M*_1_ and *M*_2_ across four species (*S*_1_ − *S*_4_) and align each family of sequences. (Third row) We acquire the joint phylogenetic tree of the two families of sequences. For each pair of positions, we place the joint sequences on the leaves of the tree as the observed states of the CTMP. The conditional probability of interval *t* is given by *e^Qt^.* (Fourth row) The joint likelihood of a CTMP along a tree is the product of prior and conditional probabilities. The marginal likelihood of each pair of aligned positions is obtained by summing over all possible states of internal nodes. It can be efficiently evaluated by dynamic programming. (Bottom row) The *log*-likelihood ratio between the coevolutionary and independent models specifies how likely the observed sequences arise from coevolution relative to the null (independent) model.

Figure 1 illustrates the procedures of evaluating the coevolutionary likelihood scores. Given the aligned sequences of two positions in different (or identical) protein domains, their joint phylogenetic tree, and the joint substitution rate matrix, we can calculate the marginal likelihood of the observed sequences on the leaves by summing over the sequence states of internal nodes. The level of fitness of the coevolutionary model to the data is measured by the *log*-likelihood ratio between the coevolutionary and independent models. For each pair of positions in the two families of aligned sequences (or one family of sequences against itself), we can calculate their *log*-likelihood ratio and mark putative coevolving position pairs.

Very often there are multiple coevolving positions between two domains (or within one single domain). To assess the likelihood score of the entire domain pair, we employ a probabilistic graphical model with variables corresponding to specific positions of the protein domains in an ancestral or contemporary species. Using a spanning tree approximation, we evaluate the joint likelihood score in terms of the pairwise and singlet likelihoods (Equation 5). The method of assessing the likelihood score of multiple coevolving pairs is novel and does not appear in our previous work [21]. Details about the coevolutionary models of position pairs and the entire domain pairs are described in Materials and Methods.

### Coevolving Protein Domains Are Functionally Coupled

The entire Pfam database of aligned protein domain sequences was downloaded [20] (April 2006 version). Overall the dataset contained 8,183 domain families. The automatically generated “full alignment'” of each domain family was chosen in order to maximize the coverage and number of sequences in the data. The topology and branch length of the phylogenetic tree for each domain family were also downloaded from Pfam.

We considered the 3,722,468 domain family pairs (12% of all family pairs) which co-appeared in no less than 20 species. Out of the 3,722,468 domain family pairs, 179,117 (4.81%) co-appear in the same proteins or share the same GO annotations (bottom level in the GO hierarchy) in more than half of the member proteins that have GO annotations. Among each domain family pair, we considered all position pairs. In total there were 1.171 × 10^11^ all-versus-all inter-domain position pairs.

**Figure 2:**
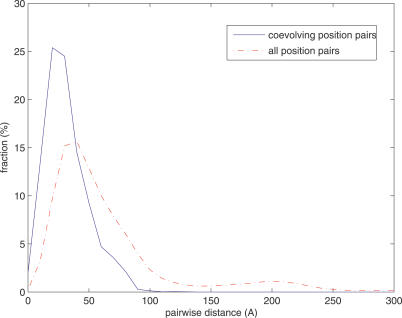
Distance Distribution of Amino Acid Residues between Two Domains Solid blue: coevolving positions. Dotted red: background.

We calculated the *log*-odds score for each position pair in each of the 3,722,468 domain pairs. We set the threshold of the *log*-odds scores to be 9.0 according to *p*-values of random CTMP simulation, false discovery rates of multiple hypotheses testing, and functionally coupled domain pairs inferred by the model. First, by randomly simulating 1 million sequences using CTMP (see Materials and Methods) we found the *p*-value for *log* likelihood ratio 9.0 is less than 6.0 × 10^−5^. Second, by randomly sampling sequences from the 3,543,351 functionally unrelated family pairs (see Materials and Methods), we plotted the dependence of false discovery rates and *log*-odds thresholds (Figure S1). Threshold 9.0 yielded the false discovery rate 33.00%. Third, when determining the threshold, there was a tradeoff between the number of functionally related domain pairs and the fraction of these “true positives” among all the positive calls (Figure S2). With threshold 9.0 the true positive rate was about 45%. In addition, the results of functional and spatial coupling in the subsequent sections are robust against the choice of threshold ≥9.0. For instance, the top 100 coevolving domain pairs (Text S1) and the distance distribution of inter-domain coevolving position pairs (Figure 2) remain unchanged when the threshold increases to 17.0.

With a threshold 9.0, we obtained 3,953 position pairs distributed over 582 domain family pairs. We then ranked the 582 inferred domain pairs according to the *log*-odds scores of the joint model for multiple coevolving positions. The sorted coevolving domain family pairs, their coevolving positions, and the *log*-odds scores are reported in Text S1.

The coevolving protein domains are highly enriched with functionally coupled domain pairs. Of the 582 domain family pairs (44.16%), 257 share proteins or bottom-level GO annotations in more than half of their members. The enrichment of functionally coupled domain pairs is more than a 9-fold increase compared to the entire dataset (4.81%). The hypergeometric *p*-value for acquiring ≥257 functionally coupled domain family pairs by randomly choosing 582 domain family pairs is less than 2.22 × 10^−174^ (see Materials and Methods). The functional coupling of the domains, however, may be a trivial consequence of many co-occurring species. To exclude this possibility, we considered the family pairs that overlapped in more than 200 species. The hypergeometric *p*-value for enrichment is less than 7.04 × 10^−45^, allowing the null hypothesis of co-occurring species to be rejected. Furthermore, 85 out of the top 100 coevolving domain family pairs are functionally coupled. The enrichment of functionally coupled domains suggests that covariation at multiple positions is a strong indicator for functional coupling.

Table 1 lists the functional categorization of coevolving domain families that are functionally coupled among the 582 inferred pairs. The majority of the domain pairs appear in the same proteins or protein complexes, whereas only a small fraction of them (26 out of 257) are in the same functional pathways. Coevolving domains primarily appear in a few classes of proteins: ribosomal proteins, RNA polymerase, metabolic enzymes, translational apparatus, bacteria conjugal transfer proteins, and virus proteins. Most of these proteins are universally essential from bacteria to human. Proteins which exhibit substantial variability, such as transcription factors, signaling proteins, and receptors are under-represented.

**Table 1:**
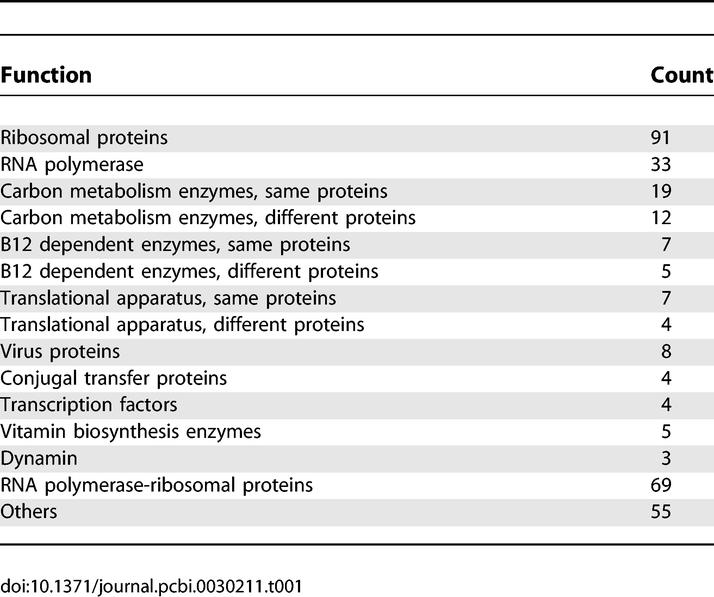
Functional Categorization of Coevolving Domain Family Pairs That Are Functionally Coupled

**Figure 3:**
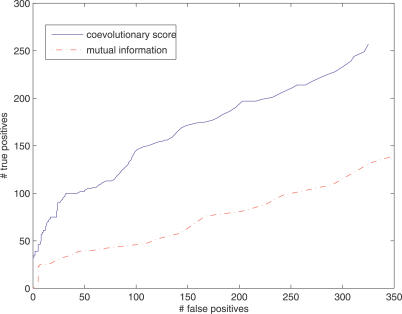
Receiver Operating Characteristic Curves of Detecting Functionally Related Domain Pairs Solid blue: coevolutionary scores. Dotted red: mutual information.

Sequence covariation without phylogenetic information can be captured by mutual information. To demonstrate the importance of phylogenetic information, we applied the same inter-domain large-scale screening using pairwise mutual information (see Materials and Methods). We counted the number of inferred domain family pairs that were functionally coupled (true positives) or not (false positives). Figure 3 shows the Receiver Operating Characteristic curves of coevolutionary and mutual information scores. The results indicate the coevolutionary model consistently outperforms the mutual information score in identifying functionally related domains. With 582 inferred domain pairs, coevolutionary scores identified 257 functionally coupled domain pairs, whereas mutual information only identified 161 functionally coupled domain pairs. In addition, the top 100 domain pairs inferred by mutual information contained only 40 functionally coupled pairs compared to 85 for coevolutionary scores.

### Coevolving Positions Are Spatially Coupled

Besides functionally coupling coevolving domains, a natural question is whether the coevolving amino acids are also spatially coupled. Of the 582 coevolving domain family pairs, 156 contain the domain pairs co-crystalized in the same proteins or protein complexes. We extracted the 196 protein/protein complex structures of the 156 coevolving domain family pairs from the Protein Data Bank [22] and mapped the coevolving positions to the amino acid residues in their PDB structures (see Materials and Methods). Figure 2 shows the distance distribution between the 4,849 coevolving position pairs and the background distance distribution of all 6,072,873 position pairs between the two domains in the same PDB structures. Clearly, coevolving position distance (solid blue) tends to be shorter and more narrowly distributed compared to the background distribution (dotted red). The *p*-value of the Kolmogorov-Smirnov test is <2 × 10^−16^. The significant difference of distance distributions suggests coevolving positions are spatially coupled. The distances of all coevolving positions in the PDB structures are reported in Text S1.

A remarkable example of the spatially coupled coevolving pair is between position 157 of the alpha-hairpin domain (accession number PF00081) and position 61 of the C-terminal domain (accession number PF02777) in iron/manganese superoxide dismutase. This domain pair ranks 82nd on the list (see Text S1).

The amino acids at positions PF00081–157/PF02777–61 exhibit strong covariation between NF and FQ (N: asparagine, F: phenylaninine, Q: glutamine, see Figure S3). Strikingly, the distances between the two positions in 13 out of the 14 homologous proteins are less than 4Å, suggesting their physical interactions.

**Figure 4:**
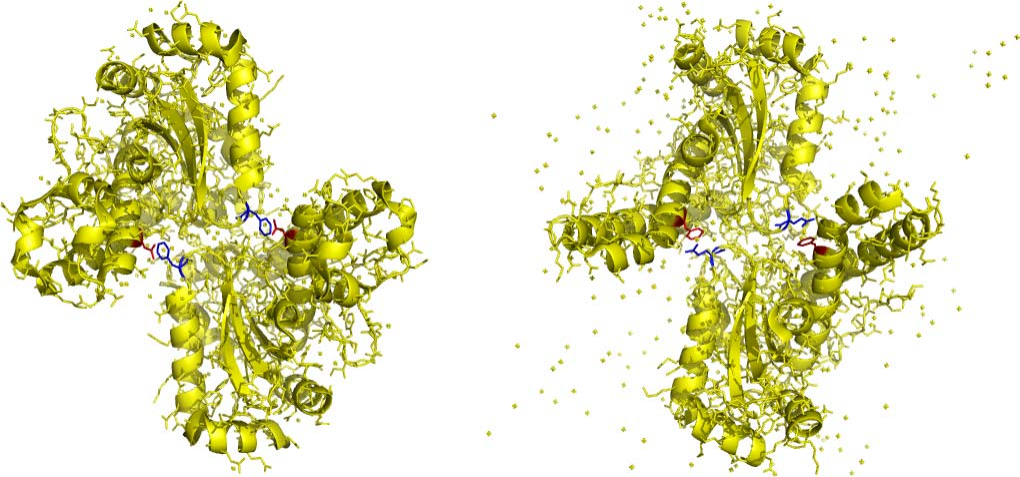
Coevolving Positions in Fe/Mn Superoxide Dismutase Left: cyanobacteria. Right: human. Coevolving positions from the two domains are marked by red and blue, respectively.

Figure 4 shows the structures of superoxide dismutase proteins in cyanobacteria (*Anabaena sp.*, PDB id 1gv3, [23]), and human (PDB id 1ap5, [24]) and marks the coevolving amino acid residues. Figure 4 was generated by *PyMOL*. The two coevolving position pairs (identical in sequence) link the two subunits of the homo-tetramer. Between cyanobacteria (NF) and human (FQ), phenylaninine (F) is swapped from the C-terminal domain to the alpha-hairpin domain, and asparagine (N) is replaced by glutamine (Q) in the same amino acid group. Hence, compensatory substitution between NF and FQ is likely to occur.

Unlike PF00081–157/PF02777–61, the majority of the coevolving positions are not in direct contact: only 4.2% (203 out of 4,849) coevolving position pairs are less than 8Å apart. Sequence covariation tends to occur between multiple distant sites of two domains. In large proteins or protein complexes constituting multiple domains (e.g., RNA polymerase or ribosome), sequence covariation between positions from multiple domains also occurs. This multi-way covariation reflects the structural or functional constraints beyond direct pairwise interactions such as hydrogen bonds. Table S1 gives examples of the multiple coevolving positions and domains.

### Coevolving Positions Are at Functionally Important Sites

The spatially distant coevolving positions may reflect certain structural or functional constraints of the entire proteins/protein complexes (e.g., [8,14,25]). To verify the functional importance of coevolving positions, we examined the coevolving positions from 25 proteins or protein complexes that were derived from the top 100 family pairs and had known structures. Intriguingly, the functionally important sites in about half of these proteins/protein complexes examined (13 out of 25) either overlapped or were near (≤10 Å) coevolving positions. Table 2 shows the functional sites near or located at the coevolving positions in the 13 proteins.

**Table 2:**
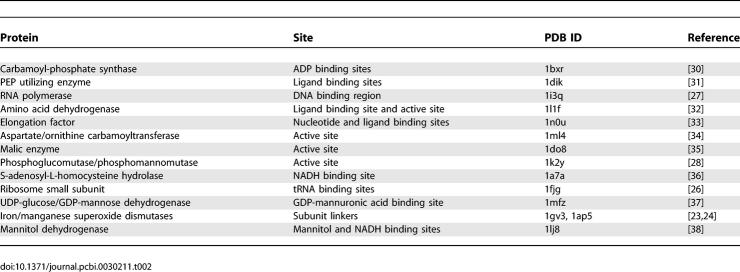
Functional Sites Overlapped/Near Inter-Domain Coevolving Positions

We use four examples to illustrate the spatial relations between inter-domain coevolving positions and functional sites of proteins.

**Figure 5:**
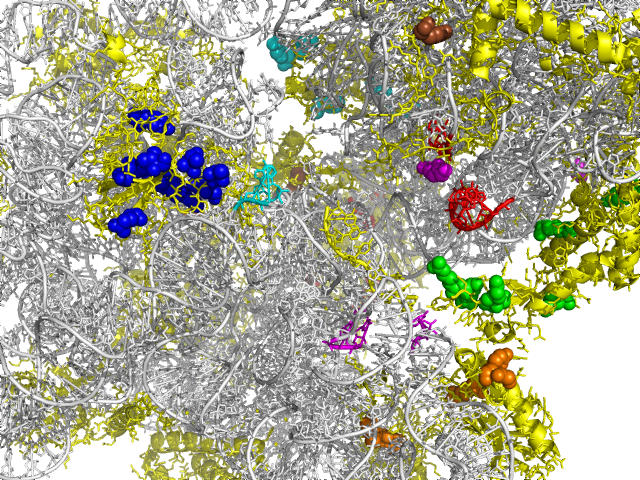
Coevolving Positions and Functional Sites in Ribosome Small Subunit Colored spheres: coevolving positions from different domains. Red ribbon: P-site. Cyan ribbon: A-site. Magenta ribbon: E-site.

There are 43 coevolving positions from ten protein domains in the 30S ribosomal subunit. Ribosomes synthesize proteins by binding tRNAs at three sites: the P (donor) site, the A (acceptor) site, and the E (exit) site. Figure 5 marks the coevolving amino acid residues (colored spheres) and the 16S rRNA nucleotides of the tRNA binding sites (colored ribbons) in Thermus thermophilus 30S ribosomal subunit ([26], PDB id 1fjg). Each tRNA binding site is close to some coevolving amino acid residues. Specifically, the S9 portion of the P site, the S12 portion of the A site, and the S7, S11 portion of the E site partially coincide with the coevolving positions.

There are 151 coevolving positions from ten protein domains in RNA polymerase. Figure S4 marks the coevolving positions in yeast RNA pol II ([27], PDB id 1i3q). These positions are located at the inner core of the macromolecule surrounding the cleft. This region directly binds to DNA (Figure 10 in [27]) and is structurally homologous between eukaryotes RNA Pol II and bacterial RNA polymerase (Figure 12 in [27]).

**Figure 6:**
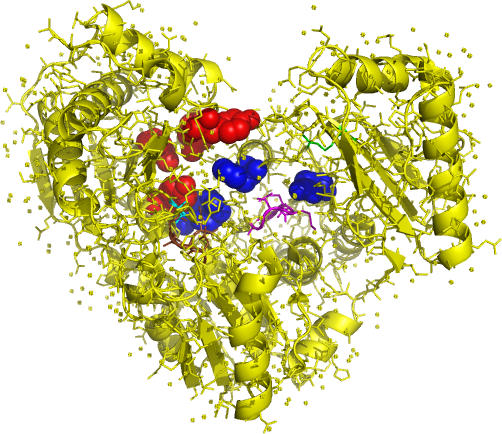
Coevolving Positions and Functional Sites in Phosphoglucomutase Red and blue spheres: coevolving positions from the two domains. Cyan ribbon: active site. Magenta ribbon: sugar binding loop. Brown ribbon: metal binding loop. Green ribbon: phosphate binding site.

There are eight coevolving positions from two protein domains in phosphoglucomutase, an enzyme that transfers the phosphoryl group of glucose or mannose from position 6 to position 1. Figure 6 marks the coevolving positions, active sites, and ligand binding sites in Pseudomonas aeruginosa phosphoglucomutase ([28], PDB id 1k2y). All except one of the coevolving position pairs are close in protein structure. Moreover, both coevolving positions and functionally important sites are located at the crevice of the heart-shaped enzyme. Functional sites including the active site, the sugar binding loop, the metal binding loop, and the phosphate binding site are all close to the coevolving positions.

There are 16 coevolving positions from two proteins in aspartate/ornithine carbamoyltransferase, an enzyme of the amino acid synthesis pathway [29]. Six out of 16 coevolving positions are close in at least three out of seven homologous protein structures. Specifically, positions 508 in the Asp/Orn binding domain and 346 in the carbamoyl-P binding domain are in contact (distance ≤4 Å) in all seven proteins. Figure S5 marks the coevolving positions and the active site in human enzyme ([29], PDB id 1c9y). Coevolving positions partially overlap with the active binding sites.

Other functional sites overlapped with, or close to coevolving positions, include ADP binding sites in carbamoyl-phosphate synthase [30]; Mg^2+^/pyruvate and nucleotide binding sites of PEP utilizing enzyme [31]; NAD, GLU binding sites, and active site of glutamate/leucine/phenylalanine/valine dehydrogenase [32]; nucleotide and sodarin binding sites of elongation factor [33]; active site of aspartate/ornithine carbamoyltransferase [34]; active site of malic enzyme [35]; NADH binding site of S-adenosyl-L-homocysteine hydrolase [36]; GDP-mannuronic acid binding site of UDP-glucose/GDP-mannose dehydrogenase [37]; and mannitol and NADH binding sites of mannitol dehydrogenase [38]. The annotations of the coevolving sites on the PDB structures of all 25 protein families are given in Text S2.

### The Effect of Gene Duplication and Deletion

Each protein domain family has a different phylogenetic tree due to its distinct history of duplication and deletion. The coevolutionary model, however, requires a joint phylogenetic tree of the two families. To calculate the likelihood score, we have to extract a common subtree of the two phylogenetic trees that correspond to the coevolving part along the lineages of the two families. This problem is difficult due to the huge number of possible choices. A common approach to compare two distinct domain (gene) trees is to reconcile them with a common species tree: mapping each node in a gene tree to a node in the species tree. There are likely multiple paralogous domains mapped to the same species. Since domains belonging to different species are unlikely to coevolve, we only need to consider the domains in the same species as candidates of the coevolving partners. For simplicity, we also hypothesize that there is at most one pair of coevolving partners from each (ancient and contemporary) species. The problem of building a joint phylogenetic tree then becomes the problem of choosing the coevolving partners in each node of the species tree.

This problem is still difficult since there are many possible combinations of coevolving partners. We employed a heuristic to construct a joint tree of two domain families and to identify the coevolving partners in each species. The goal of this heuristic is to make the joint tree respect the phylogenetic trees of individual domain families and the species where they reside, to maximize the coverage of the species in the joint tree, and to reduce the spurious covariation from paralogous members. The heuristic is described in Materials and Methods and Text S3.

Despite the advantages of the heuristic, certain covariation from early divergence is amplified when the topology of the domain tree does not conform with the species tree. A typical example is the position pairs between many RNA polymerase and ribosomal proteins (Figure S6). The pair comprises two amino acid pair sequences denoted by 1 and 2. The apparent recurrence of sequence 1 in bacteria, plants, and algae actually arises from the early divergence between bacteria/chloroplast and eukaryotes/archaea. This covariation can be structurally and functionally important, since it reflects the difference of transcription and translation apparatus between prokaryotes and eukaryotes. However, it deviates from the original purpose of identifying recurrent covariation across lineages.

To further reduce this type of covariation, we trimmed the part of the domain tree which mismatched the topology of the species tree at kingdom level. The enrichment of functionally coupled domain pairs is similar to the untreated version: 219 out of 642 inferred position pairs and 82 out of top 100 inferred pairs were functionally coupled. Most pairs between RNA polymerase domains and between RNA polymerase and ribosomal proteins were absent in the inferred pairs. Although covariation between these domain pairs does not re-occur, it is still important. It is attributed to early divergence of life, and as described previously, maintains the structurally conserved region in RNA polymerase. The inferred domain pairs by removing covariation from early divergence are reported in Text S4.

### Intra-Domain Coevolving Positions Are Spatially Coupled and Functionally Important

Our model can also detect the coevolving positions within the same protein domains. Unlike inter-domain screening, the two amino acid residues share a common phylogenetic tree. Hence spurious covariation arising from selection of paralogous proteins does not happen.

We calculated the *log*-odds score for each intra-domain position pair of all 8,183 domain families in Pfam. With a threshold value 5.0 (CTMP simulation *p*-value <3.5 × 10^−4^), we obtained 1,444 position pairs from 110 domain families. We also calculated the *log*-odds scores of the entire domains with multiple coevolving positions and ranked the 110 domains accordingly. The sorted domains, their coevolving positions, and the *log*-odds scores are reported in Text S5.

**Figure 7:**
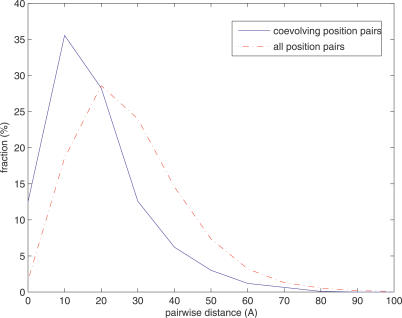
Distance Distribution of Amino Acid Residues within the Same Domain Solid blue: coevolving positions. Dotted red: background.

Two questions arising from inter-domain screening also need to be answered in intra-domain analysis. First, whether or not coevolving positions within the same domains are spatially coupled. Second, whether or not these coevolving positions overlap with or are close to functionally important sites of proteins. We extracted 401 protein structures of the 110 protein domains from the Protein Data Bank and calculated the distances between intra-domain coevolving positions. As a comparison we also calculated the distances between all position pairs in the same domain families. Figure 7 shows the intra-domain distance distributions of coevolving positions and the background. Similar to Figure 2, the distances of coevolving positions (solid blue) tend to be shorter and narrowly distributed. Both coevolving and background distributions for intra-domain positions are substantially shorter than those for inter-domain positions, as amino acids in the same domains are typically close. Yet the difference between the two distributions is still significant (*p*-value for Kolmogorov-Smirnov test <2 × 10^−16^). About 50% of coevolving positions are less than 10Å apart, whereas only about 20% of background position pairs are within 10Å. The proximity of intra-domain coevolving positions is consistent with previous studies such as [11].

To check the functional importance of coevolution, we examined the intra-domain coevolving positions from the 38 domain families that contain the position pairs with *log*-odds scores ≥8.0. The coevolving positions from 13 of these 38 domain families overlap with or are close to the functional sites of proteins. The reported functional sites are primarily active or ligand binding sites of enzymes since they are easy to identify in the literature. The coevolving positions on other proteins (such as virus coat proteins) might also carry functional information but are not evident by screening the literature. Table 3 shows the functions of intra-domain coevolving sites.

**Table 3:**
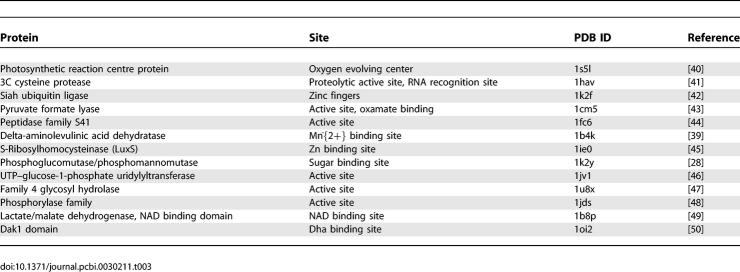
Functional Sites Overlapped/Near Intra-Domain Coevolving Positions

Two remarkable instances are domains delta-aminolevulinic acid dehydratase (PF00490) and photosynthetic reaction centre protein (PF00124). In the PF00490, there are five coevolving positions. All of them are physically close (<10Å) in all three protein structures of the domain family. These positions partially coincide with the active sites and Mn^2+^-binding sites of Pseudomonas aeruginosa dehydratase protein ([39], PDB id 1b4k). In PF00124, there are 41 coevolving positions. Some of these positions are close to the oxygen evolving center of Thermosynechococuus elongatus PSII protein ([40], PDB id 1s5l), which oxydizes water in photosynthesis. Other functional sites overlapped with, or close to, coevolving positions include proteolytic active site and RNA recognition site of 3C cysteine protease [41]; zinc fingers of Siah ubiquitin ligase [42]; active site and oxamate binding site of pyruvate formate lyase [43]; active site of peptidase family S41 [44]; Zn binding site of S-Ribosylhomocysteinase [45]; active site of UTP-glucose-1-phosphate uridylyltransferase [46]; active site of family 4 glycosyl hydrolase [47]; active site of phosphorylase family [48]; NAD binding site of lactate/malate dehydrogenase [49]; and Dha binding site of Dak1 domain [50]. The complete annotations of intra-domain coevolving sites on the PDB structures are in Text S6.

### Physical Interactions Are Not Necessarily Coevolved

Analysis in the preceding sections suggests that coevolving domains are likely to be functionally coupled, and coevolving position pairs tend to be spatially coupled and located at functionally important sites. Yet the question in the reverse direction—whether physically interacting amino acid residues are coevolved—are still not answered. Since the majority of the coevolving positions are not in direct contact, we expect the overlap set between physical interactions and coevolving positions to be small. We extracted 223,392 physical interactions from Pfam. Interactions corresponding to the same aligned positions in the domain families were collapsed together. To reduce computational time we only considered the interactions where covarying amino acid pairs (sequences that are distinct at both positions, for example, NF and FQ) comprise more than half of the members in the domain families. Only about 20% of the interactions (45,007 out of 223,392) passed this filtering criterion. We evaluated the *log*-odds scores of these 45,007 interactions. The distribution of the *log*-odds scores is centered around 0 (mean 0.209) with standard deviation 12.96. Only a small fraction of interactions (2.6%) have *log*-odds scores higher than 9.0. The results indicate covariation is not necessary for physical interactions. The majority of physical interactions are dominated by conserved sequences or sequences with unilateral changes.

## Discussion

In this study we propose a probabilistic graphical model to detect coevolution of amino acid residues and invoke large-scale screenings on all the inter-domain, intra-domain position pairs, and known domain residue interactions. Despite the large number of pairwise comparisons executed, the inferred results strongly suggest that coevolving domains and positions are functionally and spatially coupled. The majority of coevolving protein domains appears in the same proteins or shares the same functional categorization. Coevolving positions between and within protein domains are substantially closer than the background distribution. Moreover, the coevolving positions in many proteins coincide with functionally important sites such as the subunit linkers of hydrogen peroxide dismutase, tRNA-binding sites of ribosomes, and active sites of phosphoglucomutase.

Most top-ranking coevolving domain pairs are involved in fundamental functions of life: ribosomal proteins, RNA polymerase, carbon metabolism, vitamin B12 dependent enzymes, and so on. This is probably because these ancient proteins have strict structural constraints. Our model implicitly favors the case where covarying sequences maintain the structural constraints. In addition, the stringent filtering criteria of sequence covariation and a wide coverage of species required for significant scores may also exclude the lineage-specific coevolution. To detect coevolution in these variable families (such as transcription factors, receptors, and signal transduction proteins), a targeted search on more extensive sampling of a specific clade and relaxed criteria for covariation are probably required.

Since simultaneous changes of multiple nucleic or amino acids are unlikely, there must exist “transition states” between optimal configurations during evolution. These transition states may disappear in contemporary species due to their deleterious effects. In RNAs, however, we do observe non-pairing or wobbling bases in a stem. Transition states also appear in the coevolving protein domains. For example, although position pair PF00081–157/PF02777–61 in superoxide dismutase is dominated by NF and FQ pairs, there are also a few other states including FF, FE, FP, and FR. FF can serve as a transition state between NF and FQ. Intriguingly, the distance between an FR pair is 9.46 Å (PDB id 1coj), indicating the two residues are not in contact. This suggests the transition states of amino acids may be accommodated by structural variation.

Our inferred results, in agreement with previous studies of protein coevolution, reveal a fundamental difference between protein and RNA coevolution. Typically RNA coevolution occurs in disjoint nucleic acid pairs that form hydrogen bonds and are in direct contact in the 3-D structure. In contrast, there are often multiple coevolving amino acid residues in a protein, and some of them are distant in the 3-D structure. Coevolution of multiple and distant amino acid residues probably results from multiple selective constraints. Some possible explanations include the coupling of binding energy via pathways in the protein, interactions with intermediate molecules such as water, and the global constraints pertaining to the conformation of a region in a protein.

The diverse causes of protein coevolution also make validation of computational methods problematic. Unlike RNAs, there is no gold standard for a coevolutionary protein dataset. We validated the findings with indirect evidence such as the enrichment of functionally coupled domains defined by GO categories, distance distribution in protein structures, and annotations of the functions of the coevolving sites. More appropriate validation procedures and datasets may become available as we have better understanding of protein coevolution.

The existence of paralogous genes adds difficulty in analyzing coevolution. When there are multiple paralogous domains in a family, we have to assign coevolving partners from all possible combinations. Our heuristic method reduces, yet cannot eliminate, spurious covariation from paralogous families. A better algorithm of dealing with paralogous genes is needed.

To facilitate large-scale screening we applied several simplifying assumptions and procedures. First, we applied the same sequence substitution rate matrix (the Dayhoff matrix) to all the domain families. Rate variation across domains or different sites within the same domains may create spurious covariation [15]. Second, like other phylogenetic methods of detecting coevolution, the accuracy of the results generated by our model depends on the accuracy of the phylogenetic trees, which is under debate. Third, due to the difficulty of acquiring the parameters of sequence evolution and positive and negative sets of coevolution, the simulation *p*-values and false discovery rates are subject to error. Refined analysis of specific protein families are needed in order to correct the false predictions from large-scale screening.

The distribution of *log*-odds scores of known physical interactions shows most interacting amino acid residues do not possess covarying sequences, consistent with a recent finding in a yeast protein–protein interaction study [51]. The discrepancy between physical interactions and sequence covariation is attributed to many possible causes. Some interactions may be lineage-specific or have highly conserved sequences. Others may undergo unilateral changes within the same amino acid groups.

Coevolution probably only occurs in a small fraction of physical interactions. Nevertheless, we also demonstrate that coevolution manifests spatial and functional constraints other than direct interactions. Hence, the complex relations between coevolution and selective constraints are worth pursuing at a deeper level.

## Materials and Methods

### Sequence substitution model of pairwise coevolution.

The sequence substitution of a single amino acid is modeled by a CTMP [17]. Denote by *x*(*t*) the sequence composition at time *t*. *P*(*x*(*t*)) is a 1 × 20 probability vector of *x*(*t*) and follows a Markov process at an infinitesimal time interval:

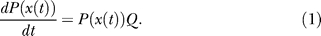
where *Q* is a 20 × 20 substitution rate matrix. Each row of *Q* must sum to 0 in order to make components of *P*(*x*(*t*)) sum to 1. In this work we used the Dayhoff matrix of amino acid substitution [52]. The transition probability *P*(*x*(*t*)|*x*(0)) at a finite time interval *t* is given by the matrix exponential *e^Qt^*, which is the solution of Equation 1:

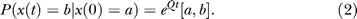



Define *x*(*t*) = (*x*_1_(*t*),*x*_2_(*t*)) as the joint state of two amino acids. The sequence substitution follows the same equation for the single-site evolution (Equation 1), but the dimensions of the probability vector (1 × 400) and the rate matrix (400 × 400) are much bigger. If two sites are independently evolved, then the joint rate matrix 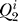 can be derived from the rate matrix of single sites [18]:

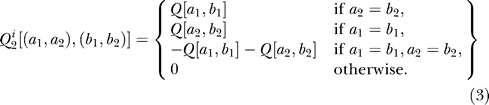




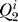
 [(*a*_1_,*a*_2_), (*b*_1_,*b*_2_)] specifies the sequence substitution rate of the independent model from state (*a*_1_,*a*_2_) to state (*b*_1_,*b*_2_). In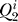 , the rate of a single amino acid change is equal to the corresponding rate in the single site rate matrix *Q*, and the rates of double amino acid changes are all zero. For example, 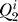 [*HR,HA*] = *Q*[*R,A*] and 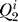 [*HR,GX*]=0. This is intuitive since off-diagonal entries of 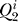 specify the transition probabilities at an infinitesimal time interval. At an infinitesimal time interval, at most one transition occurs for two independent CTMPs. Each diagonal entry of 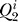 is again −1 and multiplies the sum of other entries in the same row.

A true coevolutionary model should reward transitions into the sequence states of selective advantages and penalize the transitions of opposite directions. Due to the difficulty of finding this true model, we constructed a simplified model by reweighting the entries of the independent rate matrix to penalize single transitions and to reward double transitions:

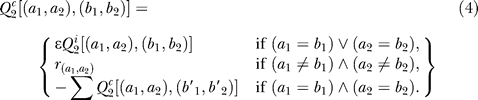



Transitions of single amino acids are penalized by multiplying a fixed number *ɛ* < 1. Transitions of double amino acids from the same state (*a*_1_,*a*_2_) are rewarded by replacing zeros with an identical quantity

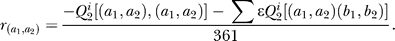



Its value forces the diagonal entries in 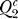 to be identical to 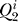 . 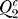 favors the sequences that have strong covariation between distinct states.

### Coevolutionary model of multiple positions.

**Figure 8:**
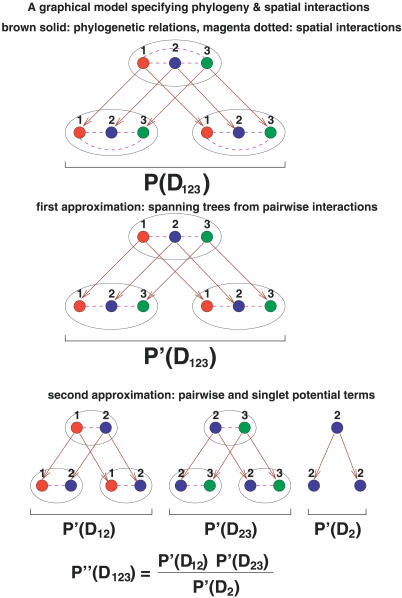
A Space-Time Model of Multiple Coevolving Positions (Top) A space-time model of three positions in three species. There are three pairwise interactions (1 2), (2 3), (3 1) in each species. *P*(*D*_123_) is the marginal likelihood of the observed sequences. (Middle) First approximation of *P*(*D*_123_). Extract the maximum spanning tree from the three pairwise interactions; ((1 2), (2 3)). *P′*(*D*_123_) is the marginal likelihood according to the approximated model. (Bottom) Second approximation of *P*(*D*_123_). Decompose the model into pairwise and singlet terms (Equation 7).

To rank the coevolving domain pairs (or single domains), we need to assess the likelihood scores which take all the coevolving positions between the two domains into account. We treated the model of all coevolving positions as a probabilistic graphical model in both space and time (Figure 8, top row). Each vertical edge on the phylogenetic tree specifies the temporal dependency between parent and child nodes, whereas each horizontal edge designates the spatial dependency between coevolving positions. These two types of dependencies create a grid-like network with many loops.

It is in general difficult to evaluate the marginal likelihood of this network. We simplified the problem by adopting two approximations. First we approximated the spatial dependency network by its maximum spanning tree (Figure 8, middle row), with the weight of each edge corresponding to its pairwise *log*-odds score. This approximation removes the loops created by horizontal edges. The likelihood of an undirected tree model can be obtained from the singlet and pairwise marginal probabilities [53,54]:

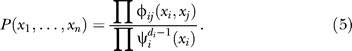
where *φ_ij_* and *ψ_i_* are marginal pairwise and singlet probabilities corresponding to edges and nodes and *d_i_* is the number of edges incident to node *i*. This formula can be obtained by assigning consistent directions to the edges and expressing the joint probability as the product of the prior probability of the root and the conditional probabilities of other nodes. The expression in Equation 5 is independent of edge direction assignments.

We assumed the conditional probability from the coevolving positions in a parent species to the same set of positions in a child species followed a form similar to Equation 5:

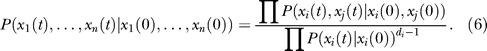
whereas *P*(*x_i_*(*t*),*x_j_*(*t*)|*x_i_*(0),*x_j_*(0)) and *P*(*x_i_*(*t*)|*x_i_*(0)) were given by the coevolutionary CTMP. Pairwise terms *φ_ij_*(*x_i_*,*x_j_*) and singlet terms *ψ_i_*(*x_i_*) in Equation 5 were replaced by conditional probabilities *P*(*x_i_*(*t*),*x_j_*(*t*) | *x_i_*(0),*x_j_*(0)) and *P*(*x_i_*(*t*) | *x_i_*(0)).

The first approximation is still intractable since it has to sum over all possible states of all coevolving positions. To further simplify the problem, we performed marginalization for each singlet and pairwise term separately and combined these terms using Equation 5. The marginal likelihoods of singlet and pairwise terms were calculated using Felsenstein's dynamic programming algorithm [55]. For instance, the marginal likelihood in the middle row of Figure 8 is approximated as

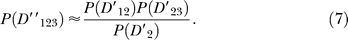
where 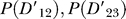 and 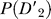


are the pairwise and singlet marginal likelihood evaluated by dynamic programming.

The marginal likelihood of the independent model is the product of the marginal likelihood for each position and can be exactly evaluated. The likelihood ratio in the bottom row of Figure 8 is

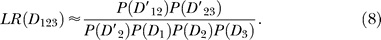



### Pfam data pre-processing.

It is costly to evaluate the coevolutionary likelihood scores. Hence we applied three filtering criteria on all 1.171 × 10^11^ inter-domain position pairs and all 8.29 × 10^7^ intra-domain position pairs. First, we discarded the sequences that contained gaps in more than half of their members. Second, we discarded the conserved sequences where one amino acid pair occurred in more than 75% of the members. Third, for each of the remaining position pairs, we identified a maximal set of covarying amino acid pairs (amino acid pairs which are distinct at both positions, e.g., NF and FQ), and counted the number of occurrences for each amino acid pair. We only considered the sequences where the maximal set of covarying amino acid pairs constituted more than 80% of the members. The first two criteria filtered out the position pairs dictated by gaps and conserved amino acid pairs. The third criterion filtered out the sequences which were expected to have low *log* likelihood ratios since the coevolutionary model (Equation 4) penalized the sequences with many unilateral changes (e.g., NF and FF). In all, 3,379,517 inter-domain position pairs and 196,198 intra-domain position pairs passed these criteria.

To further reduce computation time and error, we applied the Padé polynomial approximation for matrix exponentiation [56] and pre-computed *e^Qt^* on each branch length quantized by the following intervals: [0, 0.01, 0.02, 0.05, 0.08, 0.1, 0.2, 0.5, 0.8, 1.0]. To learn the penalty weight *ɛ*, we chose the joint sequences of the coevolving superoxide dismutase position pairs (PF00081–157/PF02777–61) as the training set and carried out a one-dimensional line search that maximizes its *log*-odds score. The optimal *ɛ* = 0.7.

### Building a joint phylogenetic tree of two domain families.

To evaluate the coevolutionary likelihood of an inter-domain position pair, a joint phylogenetic tree and representatives from each species in each domain are needed. We selected the species that contained both domains and built a binary species tree on selected species by extracting the hierarchy from the National Center for Biotechnology Information taxonomy [57]. The topology of the species tree was used as the joint tree. For each domain family, we then applied a heuristic to select one representative domain for each species that reduces spurious covariation across paralogous lineages. The idea is to label each internal node of the domain family tree as a speciation or duplication event (using a reconciliation algorithm, [58]) and to pick up an orthologous subtree that maximizes species coverage. We then incrementally updated the branch length in the mapped species tree.

The procedures of building a joint tree and selecting representatives are described in Text S3.

### Large-scale screening using mutual information.

As a comparison we calculated mutual information between the 3,379,517 inter-domain position pairs that passed the filtering criteria. Denote *x*_1_ and *x*_2_ the sequence composition of sites 1 and 2, *P*_12_(*x*_1,_ *x*_2_) the frequency of (*x*_1,_ *x*_2_) among the aligned sequences, and *P*_1_(*x*_1_) and *P*_2_(*x*_2_) the marginal frequencies of *x*_1_ and *x*_2_. The mutual information between *x*_1_ and *x*_2_ is

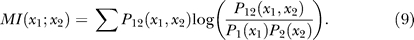
where 0log0 ≡ 0.

### Implementation of screening.

The large-scale screenings, including filtering position pairs by sequence covariation, building the joint phylogenetic tree for domain family pairs, calculating pairwise coevolutionary scores, and evaluating the joint likelihood scores of the entire domains/domain pairs were implemented in C programs and executed on Rackable Linux Cluster (2048 AMD Opteron Processors, 2.2 GHz). The total CPU time was 24,000 h for inter-domain screening, 1,600 h for intra-domain screening, and 300 h for evaluating the *log*-odds scores of known interactions. The C codes and sequence data are available per request to the corresponding author.

### Evaluating significance.

The *p*-value of the coevolutionary likelihood scores: the significance of *log*-odds scores was evaluated by random CTMP simulation. In each trial, we first randomly selected a domain family and acquired its phylogenetic tree. A subtree of 50–200 nodes was randomly extracted. We then generated the sequence pairs at leaves by simulating two independent CTMPs using the Dayhoff matrix and the selected tree. The *log*-odds score of the sampled sequence pairs was calculated. The *p*-value was the fraction of the 10^6^ random trials which yielded the *log*-odds scores ≥ threshold *θ.* The *p*-value of *θ* = 9.0 is 6.0 ×10^−5^.

The false discovery rate of coevolving position pairs: We evaluated the false discovery rate of multi-hypotheses testing using the approximation procedures in [59]. Given a *log*-odds score threshold *θ*, we calculated the false positive rate (*p*-value) by the following procedure. We uniformly selected a random domain family pair which intersected in more than 20 species and did not share the same proteins or bottom-level GO annotations in more than half of their members, and then uniformly drew two random positions. The false positive rate *P*(*θ*) is the probability of finding a position pair with *log*-odds score ≥ *θ*. Notice *P*(*θ*) is considerably smaller than the *p*-value of CTMP simulation since many position pairs were filtered out by the pre-processing procedure. Denote *m* the total number of position pairs and m(*θ*) the number of position pairs with *log*-odds scores exceeding *θ*. The false discovery rate *q*(*θ*) on threshold *θ* is approximated by

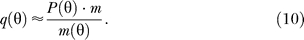



The total number of position pairs *m* = 1.17 × 10^11^. With threshold *θ* = 9.0, *p*(*θ*) = 1.114 × 10^−8^, and m(*θ*) = 3,953. Thus, *q*(*θ*) = 1.114 × 10^−8^ 1.17 × 10^11^/3953 = 0.33.

Figure S1 shows the dependency of *q*(*θ*) and *θ*. *q*(*θ*) varies from 0.33 to 0.03 as *θ* varies from 9.0 to 30.0.

The *p*-value of enrichment of functionally coupled family pair: we used the standard hypergeometric *p-*value to assess the significance of enrichment of functionally coupled domain family pairs among inferred domain family pairs. Define *N* the total number of family pairs considered, *n* the number of inferred family pairs, *K* the total number of family pairs that were functionally coupled, and *k* the number of inferred family pairs that were functionally coupled. The hypergeometric *p-*value was the probability of randomly drawing *n* from *N* pairs (without replacement) where more than *k* pairs were functionally coupled:

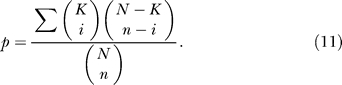



### Acquiring protein structure data and calculating residue distances.

We downloaded 196 protein structure data from the 582 inter-domain family pairs and 401 protein structures from 110 intra-domain families from the Protein Data Bank [22]. We mapped a position in a domain to an amino acid residue in its PDB structure by aligning the domain sequence and PDB sequence of each chain in the PDB using ClustalW [60]. The closest distance between the atoms from the two amino acid residues was reported. If a protein domain could be mapped to multiple chains of a PDB, then from all possible amino acid residue pairs we reported the one with the smallest distance.

## Supporting Information

Figure S1 The Dependence of False Discovery Rates on the Thresholds of *log*-likelihood Scores (1 KB PDF)

Figure S2 The Dependence of Number and Rate of True Positives on the Thresholds of *log*-likelihood Scores (3 KB PDF)

Figure S3 The Joint Phylogenetic Tree of Iron/Manganese Superoxide Dismutase Domains, PF00081/PF02777, and the Amino Acid Sequences on Positions 157/61 (25 KB PDF)

Figure S4 The Coevolving Amino Acid Pairs on the PDB Structure of RNA Polymerase (PDB ID 1i3q) (51 KB PDF)

Figure S5 The Coevolving Amino Acid Pairs on the PDB Structure of Carbamoyltransferase (PDB ID 1c9y) (36 KB PDF)

Figure S6 An Example of Spurious Covariation Due to the Mismatch between Species and Gene Trees (5 KB PDF)

Table S1 The Multiple Coevolving Domains (7 KB PDF)

Text S1 The Sorted Coevolving Domain Family Pairs, Their Coevolving Positions, and the *log*-odds Scores (285 KB TXT)

Text S2 The *PyMOL* Script Annotating the Inter-Domain Coevolving Sites on PDB Structures (84 KB TXT)

Text S3 The Description of the Heuristic for Building a Joint Phylogenetic Tree and Selecting Representatives from Species and Domain Trees (34 KB TXT)

Text S4 The Sorted Coevolving Domain Family Pairs Acquired by Removing Covariation from Early Divergence of Life, Their Coevolving Positions, and the *log*-odds Scores (272 KB TXT)

Text S5 The Intra-Domain Coevolving Positions and the *log*-odds Scores (48 KB TXT)

Text S6 The *PyMOL* Script Annotating the Intra-Domain Coevolving Sites on PDB Structures (26 KB TXT)

## Accession Numbers

The accession numbers listed in this paper from the Protein Data Bank (http://www.rcsb.org/pdb) are alpha-hairpin iron/manganese superoxide dismutase domain, position 157 (PF00081), C-terminal iron/manganese superoxide dismutase domain, position 61 (PF02777), delta-aminolevulinic acid dehydratase (PF00490), and photosynthetic reaction centre protein (PF00124).

## Abbreviations

A: ribosome acceptor site
CTMP: continuous-time Markov process
E: ribosome exit site
F: phenylaninine
N: asparagine
P: ribosome donor site
PDB: protein data bank
Q: glutamine
