# Supplementary figures and images for: Detecting Coevolution in and among Protein Domains
Source: PLoS Comput Biol. 2007 Nov 2;3(11):e211. doi: 10.1371/journal.pcbi.0030211 (PMC2098842; doi:10.1371/journal.pcbi.0030211)

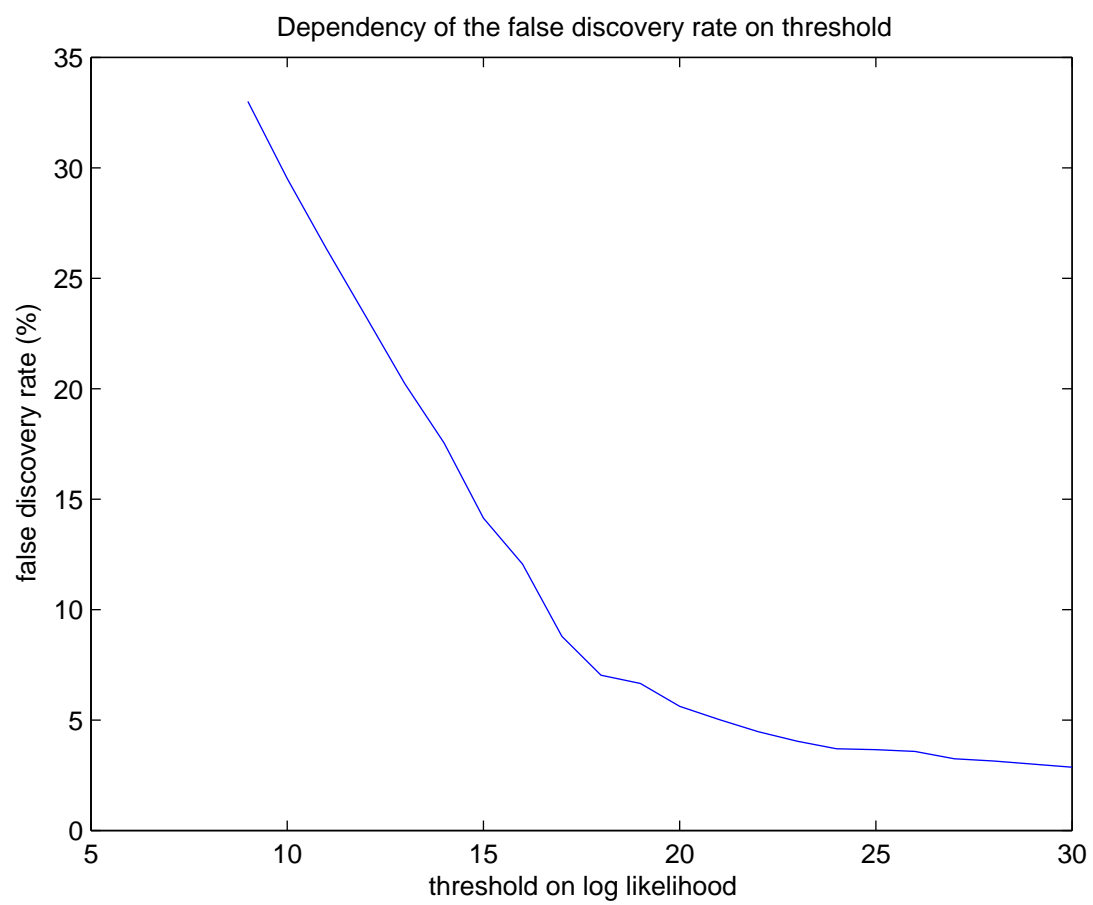

Supplement: Figure S1 — (1 KB PDF) [file pcbi.0030211.sg001.pdf]

Functionally related domains inferred from coevolutionary scores

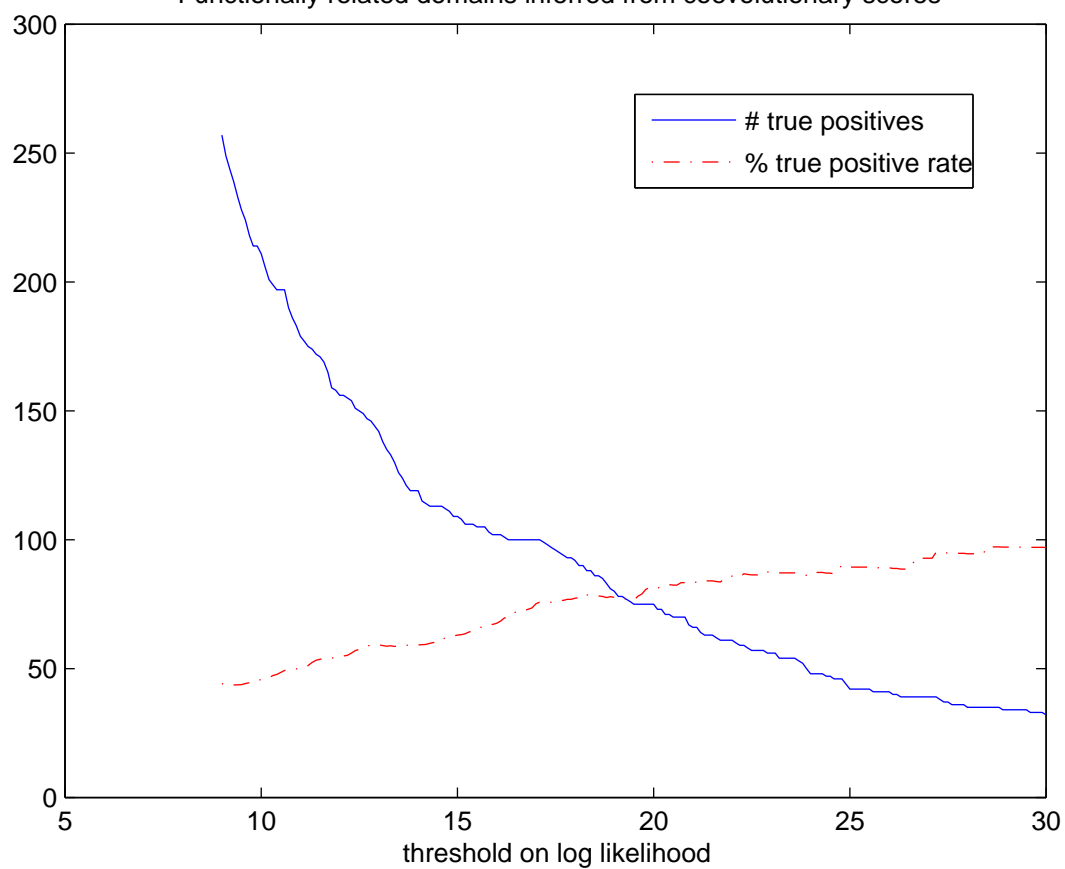

Supplement: Figure S2 — (3 KB PDF) [file pcbi.0030211.sg002.pdf]

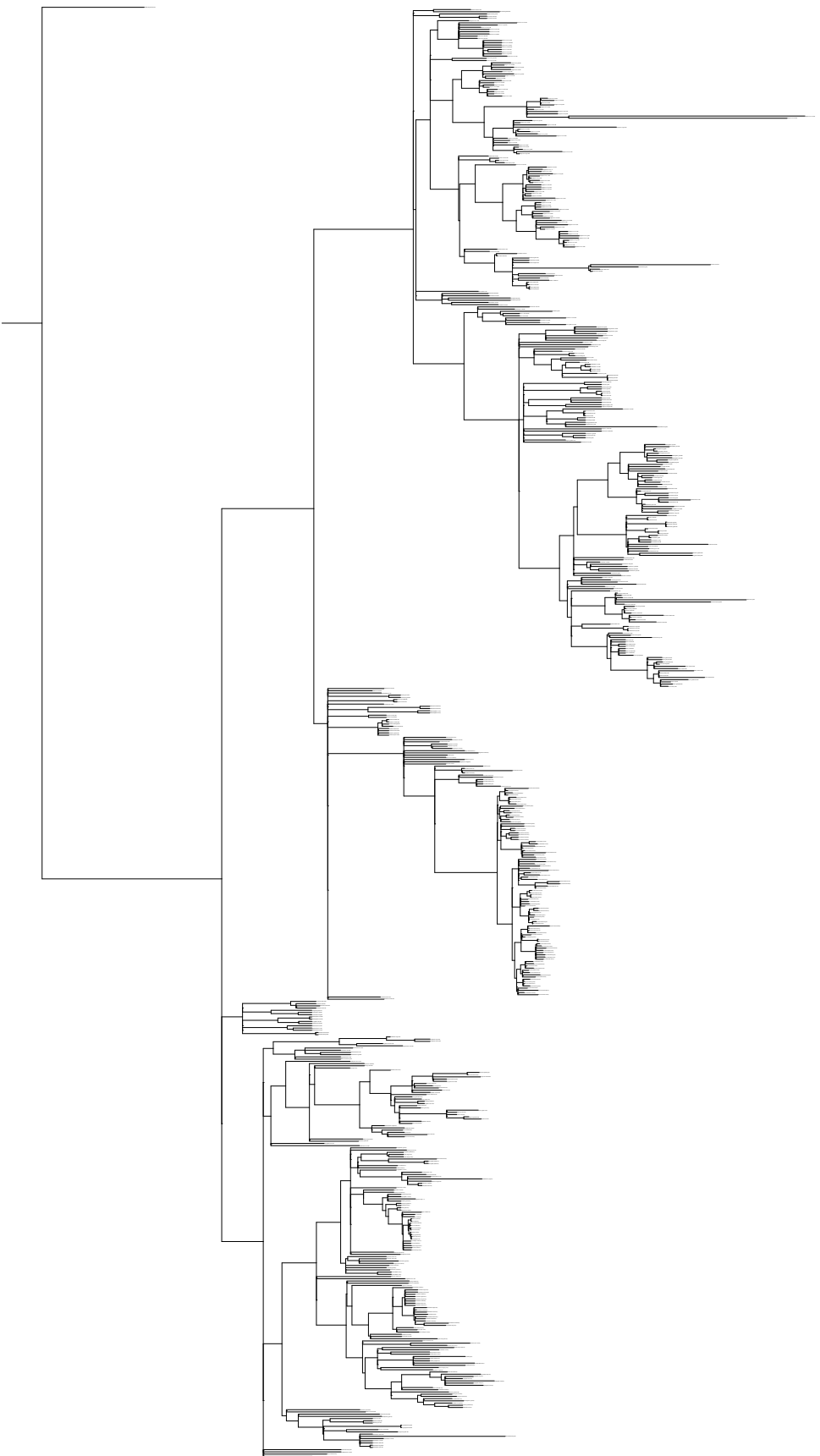

Supplement: Figure S3 — (25 KB PDF) [file pcbi.0030211.sg003.pdf]

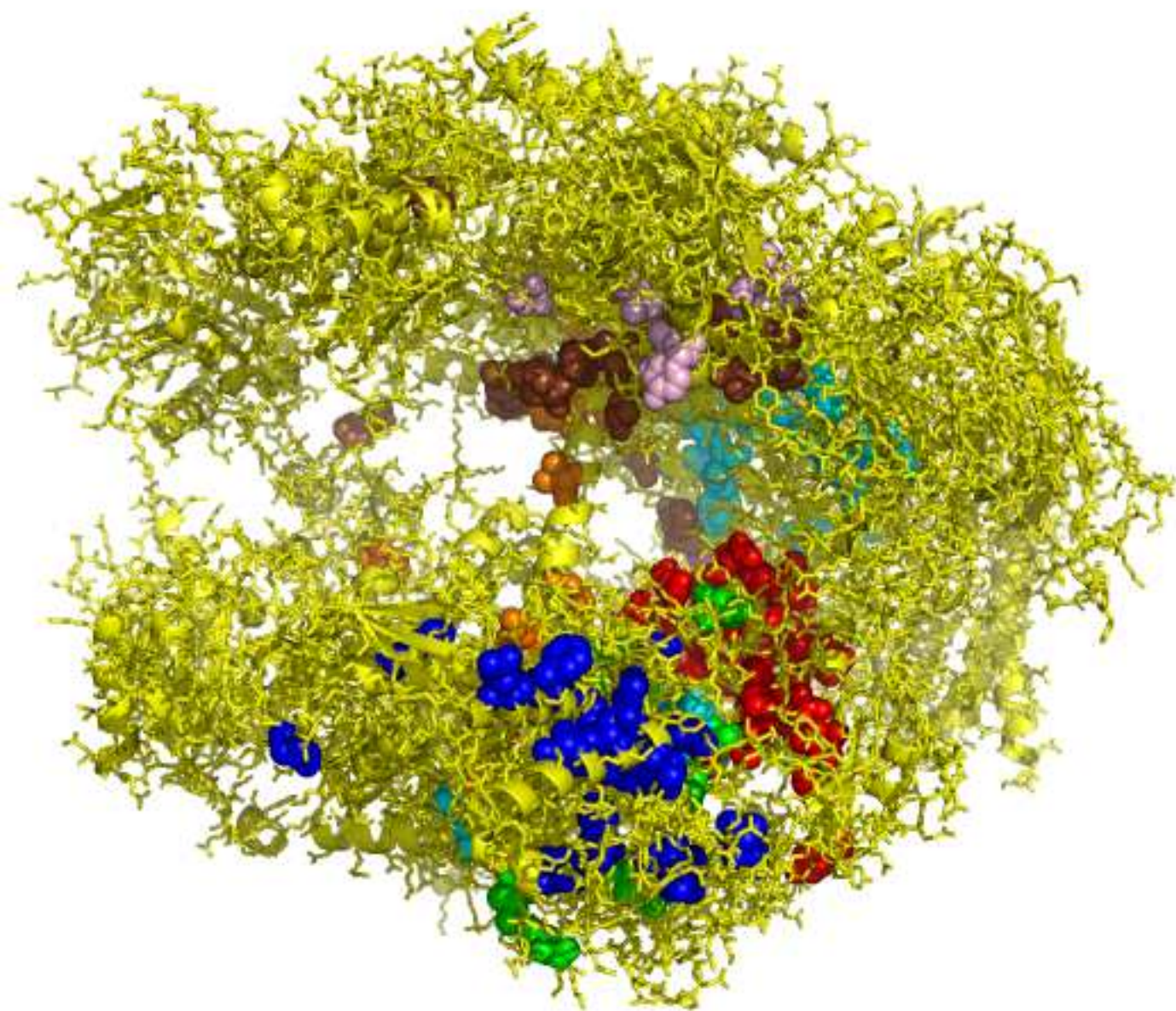

Supplement: Figure S4 — (51 KB PDF) [file pcbi.0030211.sg004.pdf]

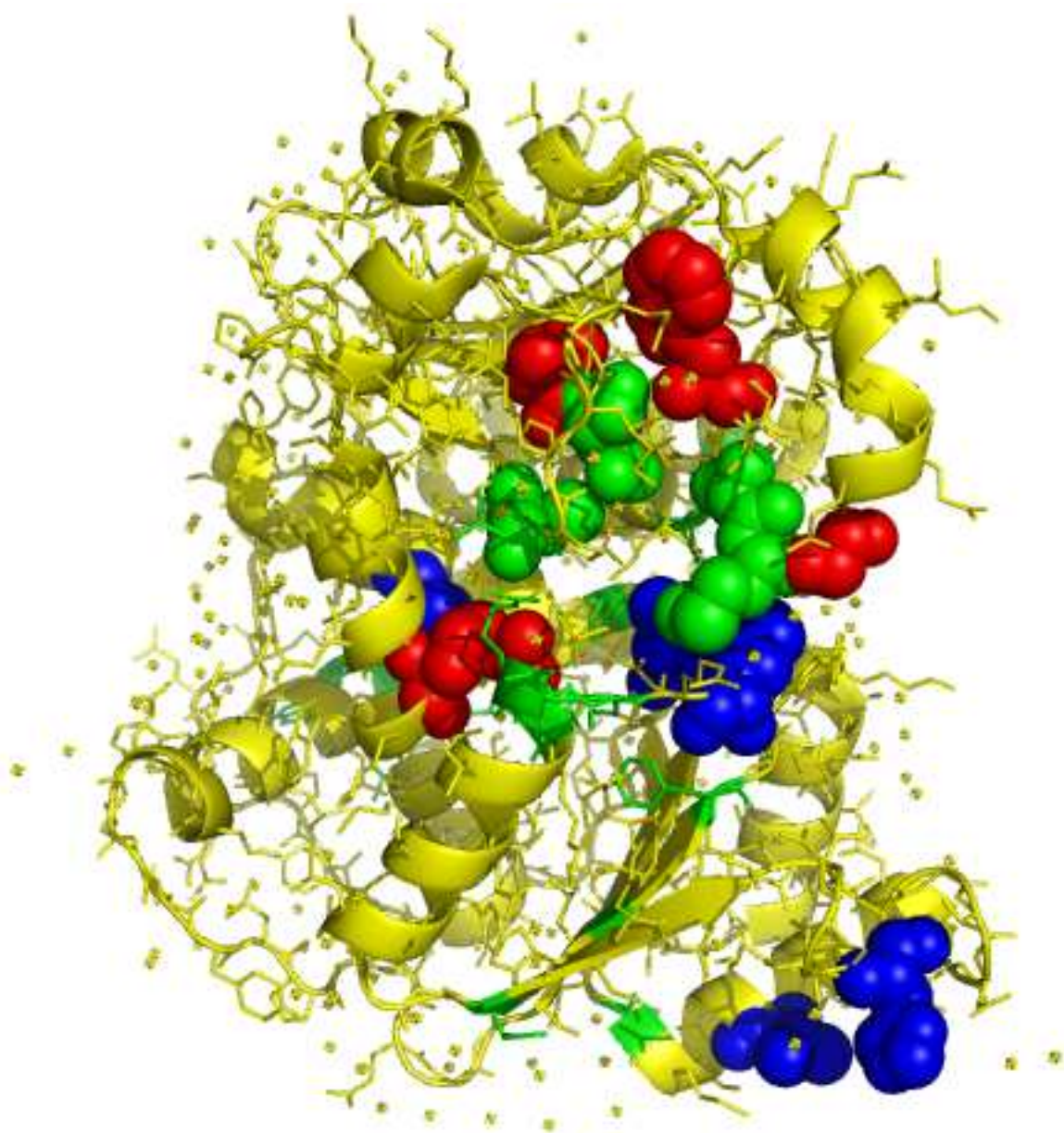

Supplement: Figure S5 — (36 KB PDF) [file pcbi.0030211.sg005.pdf]
