# Supplementary material for: Detecting Coevolution in and among Protein Domains
Source: PLoS Comput Biol. 2007 Nov 2;3(11):e211. doi: 10.1371/journal.pcbi.0030211 (PMC2098842; doi:10.1371/journal.pcbi.0030211)
Supplement: Figure S6 — (5 KB PDF) [file pcbi.0030211.sg006.pdf]

Covariation due to the mismatch of gene tree and species tree

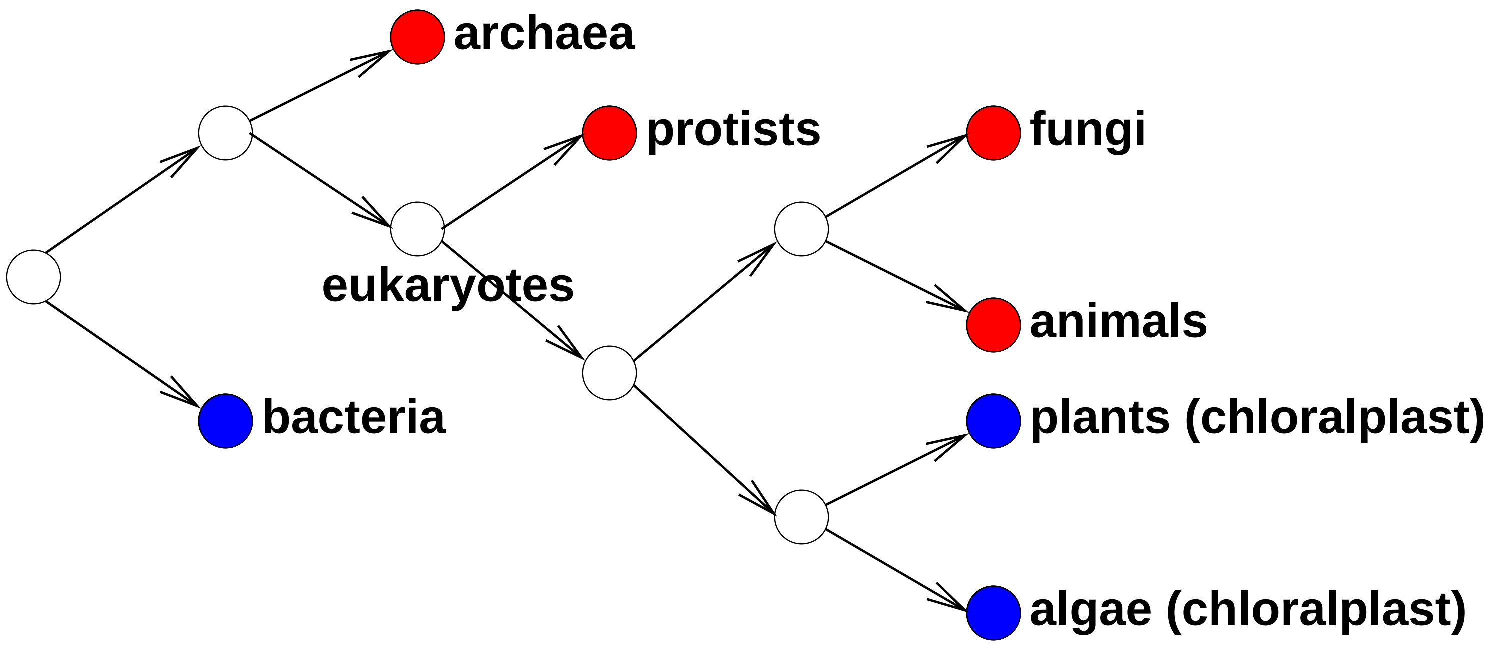

sequence evolution on species tree.
sequence pair 1 (blue) appears in bacteria, plants and algae.
sequence pair 2 (red) appears in archaea, non−green eukaryote.
transition between 1 and 2 occurs twice.

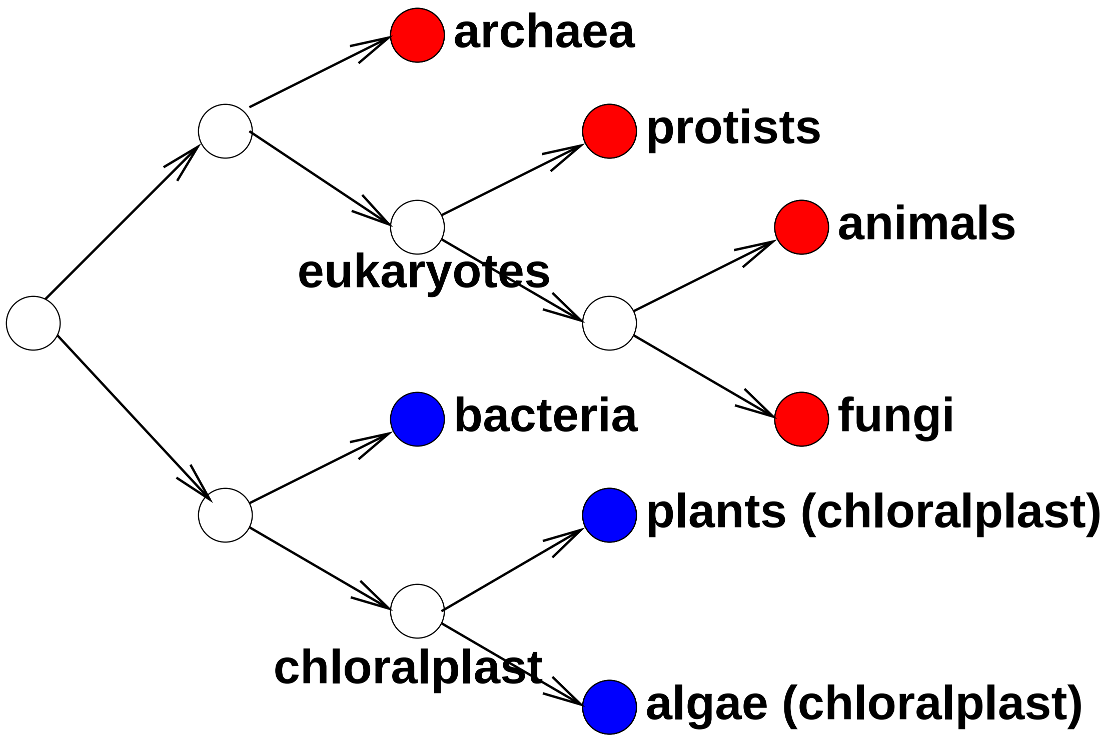

sequence evolution on gene tree.
sequence pair 1 (blue) appears in bacteria and chloralplast.
sequence pair 2 (red) appears in eukaryotes and archaea.
transition between 1 and 2 occurs once.
