# Supplementary material for: Detecting Coevolution in and among Protein Domains
Source: PLoS Comput Biol. 2007 Nov 2;3(11):e211. doi: 10.1371/journal.pcbi.0030211 (PMC2098842; doi:10.1371/journal.pcbi.0030211)
Supplement: Table S1 — (7 KB PDF) [file pcbi.0030211.st001.pdf]

function	count
ribosomal proteins	91
RNA polymerase	33
carbon metabolism enzymes, same proteins	19
carbon metabolism enzymes, different proteins	12
B12 dependent enzymes, same proteins	7
B12 dependent enzymes, different proteins	5
translational apparatus, same proteins	7
translational apparatus, different proteins	4
virus proteins	8
conjugal transfer proteins	4
transcription factors	4
vitamin biosynthesis enzymes	5
dynamin	3
RNA polymerase-ribosomal proteins	69
others	55
