# Supplementary material for: Detecting Coevolution in and among Protein Domains
Source: PLoS Comput Biol. 2007 Nov 2;3(11):e211. doi: 10.1371/journal.pcbi.0030211 (PMC2098842; doi:10.1371/journal.pcbi.0030211)

In this supplement we describe a heuristic of building a joint phylogenetic tree and selecting representatives from two domain family (gene) trees and their species tree.

Building a joint phylogenetic tree and selecting representatives

We applied a heuristic to build a joint phylogenetic tree of two domain families and to select representatives from the paralogous domains in each species. The goal of this heuristic is to make the joint tree respect the phylogenetic trees of individual domain families and the species where they reside, to maximize the coverage of the species in the joint tree, and to reduce the spurious covariation from paralogous members.

Find the overlapped species of the two domain families

We only consider the species that contain at least one member in both domain families.

Build a joint phylogenetic tree topology

We extract the subset of the NCBI taxonomy on the selected species and build a binary species tree. If one taxonomic group contains multiple subgroups, create dummy nodes between the two levels to force the tree to be binary. The topology of this species tree is used as the joint phylogenetic tree. An example of a species tree and a gene tree is illustrated in Figure 1.1.

Apply reconciliation to species and domain trees

We apply a reconciliation algorithm (Zmasek and Eddy 2001) to map each node in a gene (domain) tree to a node in the species tree, and to annotate each internal node in the gene tree as a duplication or speciation event. A leaf node of a gene tree is mapped to the a leaf node in the species tree where it belongs. Following a reverse topological order, the algorithm recursively maps an internal gene node to a species node which respects the local structure of the species subtree rooted at the internal node. An internal gene node is labeled as duplication if it maps to the same species node as any one of its children. An example of the reconciled tree is illustrated in Figure 1.2.

Selecting representatives from paralogous members

To apply the coevolutionary model, for each domain family we have to select one representative from each species. We select the representatives according to two criteria: to maximize the coverage of species and to choose members under the same orthologous lineage. The former is needed in order to accommodate sufficient covarying sequences. The latter is needed in order to reduce the spurious covariation between paralogous members. We first keep all the genes (domains) which do not contain paralogs in a species. We then apply a recursive algorithm to pick up a specific orthologous lineage that maximize species coverage. Starting from the root of the reconciled gene tree, recursively retaining or prunning the subtrees under each internal node. If an internal node is labeled as speciation, then keep both branches and proceed to its both children. If it is labeled as duplication, then count the number of species covered under each child and proceed only to the child that has larger coverage. An example of the selected representatives and the orthologous lineage are marked as red in Figure 1.2. An overlayed gene tree on the species tree is illustrated in Figure 1.3.

Determining the branch length

To determine the branch length in the joint tree, we first map the branch length in each gene tree to the branch length in the species tree. Reconciliation maps each edge in the gene tree to either an edge, a path, or a node in the species tree. Following the traversing order of a gene tree, we incrementally update the branch length in the mapped species tree. If an edge e_g in the gene tree is mapped to an edge e_s in the species tree, then add the branch length of e_g to the branch length of e_s. If e_g is mapped to a path $e_{s1}, \cdots, e_{sk}$ in the species

tree, then add the branch length of e_g to the branch length of e_{sk}, the last edge of the path. If e_g is collapsed to a node v_s in the species tree, then find the fist descendant of v_s which is not a collapse of e_g, and add the branch length of e_g to the edges from the parent of v_s to v_s in the species tree. We then calculate the average of the species tree branch length acquired from both domain (gene) trees and use the averaged branch length as the branch length of the joint tree.

Fig. 1. The procedures of reconciliation and selecting representatives

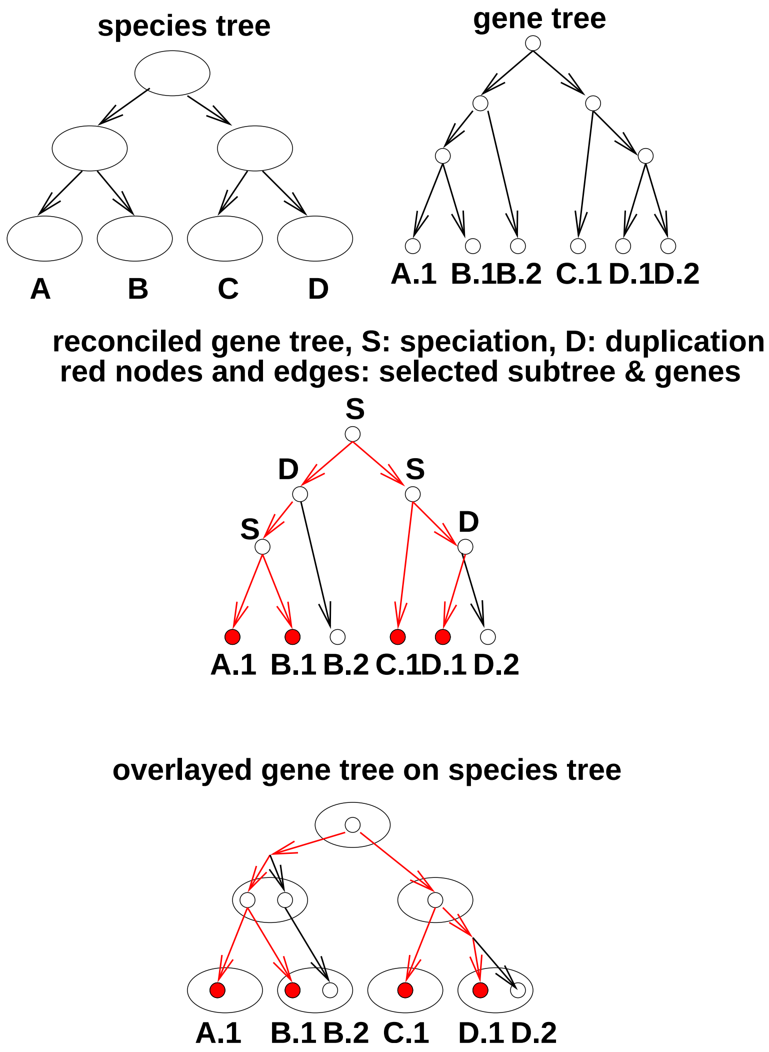

Supplement: Text S3 — (34 KB TXT) [file pcbi.0030211.sd003.pdf]
